# 

**Published:** 1884-10-20

**Authors:** 


☞ Friends, send in the amounts due, and oblige the managing editor, R. C. WORD.

### EDITORIAL NOTICES.

ST. LOUIS MEDICAL JOURNAL.—See the advertisement of this excellent journal in this issue. It clubs with our journal at low rates.

DR. HARTER'S IRON TONIC.—This preparation, an advertisement of which is in this journal, has proven to be an excellent combination. It contains iron, calasaya bark, phosphorus, coca and viburnum.

DEATH OF DR. HOUSTON.—We are pained to hear that our young friend, Dr. Thos. F. Houston, formerly of Atlanta, is no more. He died, recently, at his home in Clarksville, Ga.

SUPPOSITORY CO., CHICAGO, ILL.—The method of medication by suppositories is becoming quite common. See the advertisements in this issue of the Chicago Suppository Co., including Pencils, Bulbs, Suppositories, etc., for nose urethra, lachrymal duct, etc.

PROF. GASTON.—The able and interesting address of Prof. Gaston on entering the Chair of Surgery in the SOUTHERN MEDICAL COLLEGE on the 8th inst. was listened to by the class and a large number of citizens and professional hrethren with marked interest. In a future number we expect to publish the address. Dr. Gaston's ability as a teacher is unmistakable, and his extensive experience as a surgeon will give an additional impetus to the growing popularity of the SOUTHEEN MEDICAL COLLEGE.

### *RECEIPTED.*

Receipts will appear in our next.

